# Modification of surface topographies to inhibit candida biofilm formation

**DOI:** 10.1371/journal.pone.0308705

**Published:** 2024-10-28

**Authors:** Mohammad Islayem, Abdulrahman Agha, Mohammad T. Al Bataineh, Mohammad Saleh Bataineh, Anas Alazzam

**Affiliations:** 1 Department of Mechanical & Nuclear Engineering, Khalifa University of Science & Technology, Abu Dhabi, United Arab Emirates; 2 Department of Basic Medical Sciences, Faculty of Medicine, Yarmouk University, Irbid, Jordan; 3 Department of Mathematics, University of Sharjah, Sharjah, UAE; 4 Department of Mathematics, Yarmouk University, Irbid, Jordan; 5 System on Chip Lab, Khalifa University of Science & Technology, Abu Dhabi, United Arab Emirates; University of Pennsylvania, UNITED STATES OF AMERICA

## Abstract

The rise of infections associated with indwelling medical devices is a growing concern, often complicated by biofilm formation leading to persistent infections. This study investigates a novel approach to prevent *Candida albicans* attachment on the surface by altering surface topography. The research focuses on two distinct surface topographies: symmetry (squares) and non-symmetry (lines), created through a direct laser photolithography process on a Cyclic olefin copolymer (COC) surface. The wettability of these patterned surfaces was then examined immediately after fabrication and plasma treatment to mimic the sterilization process of indwelling devices through UV plasma. The results reveal directional wettability in the line pattern and size-dependent wettability in both square and line patterns. *Candida albicans* were cultured on these surfaces to assess the efficacy of the topography in preventing biofilm formation. The study demonstrates that symmetry and non-symmetry pattern topography inhibit biofilm formation, providing a promising strategy for mitigating Candida-associated infections on medical devices. The research sheds light on the potential of surface modification techniques to enhance the biocompatibility of medical devices and reduce the risk of biofilm-related infections.

## Introduction

Modern medical practices frequently include using indwelling medical devices. Infections related to catheters constitute a significant contributor to illness and death in hospitalized patients, primarily attributed to the presence of microbial biofilms on catheter surfaces in most cases [1]. Infections related to these devices usually start with the growth of microorganisms and eventually lead to the formation of biofilms [1]. Biofilms are groups of attached microorganisms surrounded by a matrix rich in polysaccharides that are known for their ability to resist antimicrobial medications [2]. Candida species are significant nosocomial pathogens and are frequently associated with indwelling medical device infections [2, 3]. For example, Chandra et al. analyzed the biofilms created by *Candida albicans* on silicone elastomer (SE), a prevalent catheter material [4]. Imamura et al. created and analyzed *in vitro* models to investigate the biofilm formation on different soft contact lenses with three strains of *C*. *albicans* [5]. Their findings showed that all *C*. *albicans* isolates developed biofilms on the lenses under examination, and the architecture of the biofilms varied depending on the lens type. Candida species are recognized for their capability to create biofilms on urinary catheters and intrauterine devices as well [6]. Auler et al. documented the retrieval of *C*. *albicans* from biofilms developed on the surfaces of intrauterine devices (IUDs) in individuals experiencing recurrent vulvovaginal candidiasis [7].

The preferred treatment method is often removing the infected device, which is challenging or impossible in many instances and may result in additional complications [1]. Hence, the capacity of Candida species to create biofilms plays a role in both surface-level and widespread candidiasis, highlighting the limitation of existing antifungal treatments in effectively curing these diseases [8]. Several surface modification techniques have been proposed to influence biofilm attachment and overcome this issue. One method that has been studied is the microscale patterning of surfaces to alter their topography, where surface topography and wettability are a critical factor that influences the attachment of cells [9]. Biofilm development, along with various other biological processes, relies on the interaction of cells with surfaces, where cell adhesion plays a pivotal role in facilitating this contact [10]. The wettability of a surface affects the surface’s capacity to attract liquids, thereby influencing the adherence of cellular proteins and significantly shaping cell behavior [11]. Studies suggest cells generally adhere to surfaces with contact angles ranging from 40° to 70° [12]. Chung et al. [13] investigated the effect of incorporating a rough pattern onto polydimethylsiloxane (PDMS) elastomer through photolithography, fabricating the Sharklet AF™ topography design. Over 21 days, *Staphylococcus aureus* cultures were grown on smooth and patterned surfaces. The patterned surface exhibited a noticeable decrease in biofilm formation compared to the smooth surface from day 0 to day 21. Similarly, Reddy et al. [14] utilized three different designs of the Sharklet AF™ colonized with a uropathogenic strain of *E*. *coli*. Results showed a reduction in the colony-forming unit in all three designs, where it was the highest with the 2x2 μm Sharklet in a tryptic soy broth.

Material selection in biological assays plays a crucial role in determining the effectiveness and outcomes of the tests. These substrates can significantly affect essential factors like cell viability, growth, and the binding of proteins and drugs [15]. Cyclic Olefin Copolymer (COC) has gained recognition as a noteworthy material in biotechnology owing to its excellent properties. Mainly due to its consistently low or negligible extractable properties, making it exceptionally compatible with biological systems [16]. The effect of surface wettability on *Candida albicans* biofilm formation on COC substrates was studied in our previous work [11]. The patterned wettability was created using photolithography followed by plasma treatment, which reduced COC’s water contact angle from 110° to 40°. The biofilm formation was compared for both patterned and unpatterned surfaces. The results indicated that the hydrophilic surfaces exhibited higher biofilm formation than the hydrophobic surface. However, as evident from the reported literature, it’s essential to consider the impact of surface topography and wettability on cell behavior and surface energy.

This work tested a new approach to reduce *C*. *albicans* biofilm attachment on indwelling devices by altering the surface topography. Two different topographical patterns, symmetry and non-symmetry, with varying sizes, were fabricated on COC substrates. The fabrication involves patterning through photolithography and swelling caused by immersing the COC in an alkane hydrocarbon [17]. The wettability of all designs was studied before and after plasma treatment. The *C*. *albicans* were cultured on both patterned and unpatterned surfaces after plasma treatment to mimic the same conditions used in indwelling devices after sterilizing them using UV plasma.

## Materials and methods

### Materials

COC film (thickness: 175 μm, TOPAS, Germany), COC polymer substrate (thickness: 1.5 mm, TOPAS, Germany), n-decane (Sigma-Aldrich, USA), Microposit S1813 positive photoresist (micro resist technology, Germany), isopropanol (IPA) (absolute, > 99.8%, Sigma- Aldrich, USA), ethanol (absolute, >99.8%, Sigma-Aldrich, USA) and deionized water (DI) were used in this study as obtained.

### Swelling-based topography

**Fig 1:**
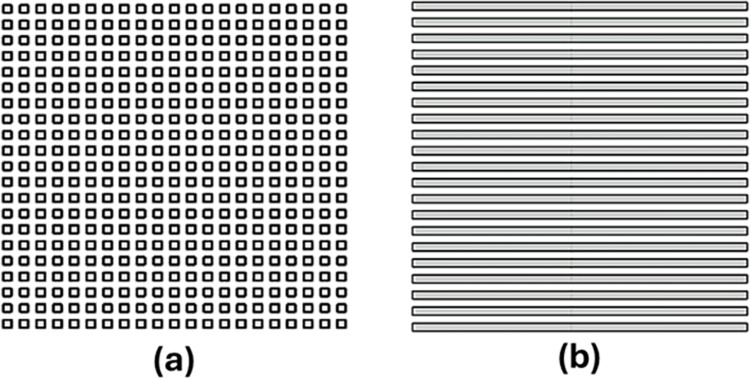
Schematic diagrams illustrating the two studied topographies. (a) Squares design (symmetrical) and (b) Lines design (non-symmetrical).

**Fig 2:**
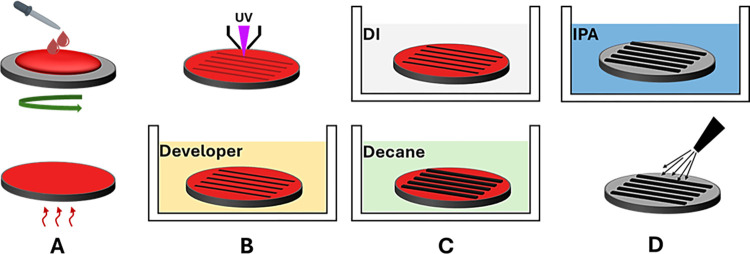
Swelling-based topography fabrication process, (A) photoresist layer deposition and heating, (B) patterning the designs using direct laser photolithography and the development process, (C) washing the substrate with DI water and then immersing it into decane, (D) using isopropanol to remove the remaining photoresist.

**Fig 1** presents schematic diagrams of the two designs considered in this study. The schematic of the swelling-based fabrication process is shown in **Fig 2**. Firstly, appropriately sized films of COC (175 μm thickness) were obtained by cutting them from a larger COC foil. The COC film surface was deposited with Microposit S1813 photoresist, then spin-coated and heated at 90°C for 2 minutes (**Fig 2A**). Next, the COC film with a photoresist layer was patterned by direct laser writer photolithography (KLOE 650) to attain the intended shape designed in AutoCAD. Afterward, the COC film was immersed in a suitable developer to remove the exposed photoresist. The COC is then removed from the developer, washed with DI water, dried, and then immersed in an alkane hydrocarbon (Decane) for 1 hour, causing the exposed areas to swell. The remaining photoresist was then removed using isopropanol. As a result, the exposed surface of the substrate expanded in volume with the desired pattern. Finally, the COC substrate underwent a thorough cleaning process in a DI water bath, followed by drying using compressed nitrogen, and then sanitized with isopropanol.

### Contact angle measurements

A contact angle goniometer (Ossila L2004A1) was utilized to measure the contact angle of the surfaces. A pipette deposited five droplets of 4 μl volume of water on each surface. Subsequently, the droplets were photographed and analyzed using Ossila software. The five droplets were taken at different locations on the substrate to ensure accurate results. Both the mean and standard deviation values were calculated and reported.

### Plasma treatment

After analyzing the wettability of the surface with patterned topographies, the entire surface underwent oxygen plasma treatment (PDC-002 equipment from Harrick Plasma, USA). The treatment lasted for 30 seconds under a pressure of 700 mTorr and a power of 7.2 W. Subsequently, contact angle measurements were conducted again after the plasma treatment.

### Strains and cultural conditions

Throughout this investigation, the wild-type *C*. *albicans* strain DK318 was utilized under standard growth conditions that do not stimulate filament formation [18]. The growth medium employed was YEPD, containing 2% yeast extract, 2% peptone, and 1% glucose, maintained at 30°C. The biofilm formation assay involved cultivating the wild-type strain in YEPD medium at 30°C until reaching an OD600 of around 4.0 [11]. This culture was then diluted at a 1:10 ratio into 50 ml of pre-warmed YEPD medium supplemented with 10% fetal bovine serum (FBS) at 37°C. The resulting cultures were agitated at 200 rpm for 24 hours [19]. For imaging, cultures were collected at the 24-hour mark, fixed using 4.5% formaldehyde, and subsequently washed twice with 1× phosphate-buffered saline (PBS) to remove any residual YEPD before imaging [20].

### Biofilm formation

A suspension of wild-type *C*. *albicans* cells (10^7^ cells/ml) was applied onto COC surfaces with patterned surface topography and unpatterned COC one hour after plasma treatment. The strain was cultured overnight in a YEPD medium under strong filament-inducing conditions. The YEPD medium was supplemented with 10% serum at 37°C and in YEPD at 30°C without serum as a control. The fungal strain was then applied to the surfaces placed in a tissue culture plate.

## Results

### Square array design (Symmetrical topography)

**Fig 3:**
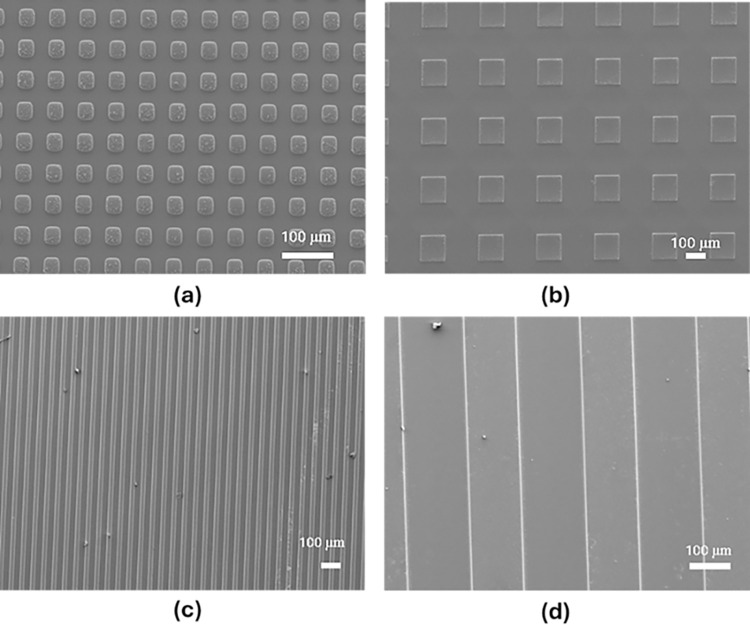
SEM images showcasing the patterned surfaces. (a) Squares with 30 μm dimensions, (b) Squares with 150 μm dimensions, (c) Lines with 30 μm length and spacing, and (d) Lines with 150 μm length and spacing.

The first design features symmetrical topography and is constructed using a square array layout, with each square’s size matching the spacing between them. This design was realized in seven sizes (10, 30, 50, 70, 100, 150, and 250 μm squares). **Fig 1A** presents a schematic diagram of the square design topography, while **Fig 2** illustrates the fabrication method. **Fig 3A** and **3B** show the SEM images for the 30 μm and 150 μm spacings, respectively.

**Fig 4:**
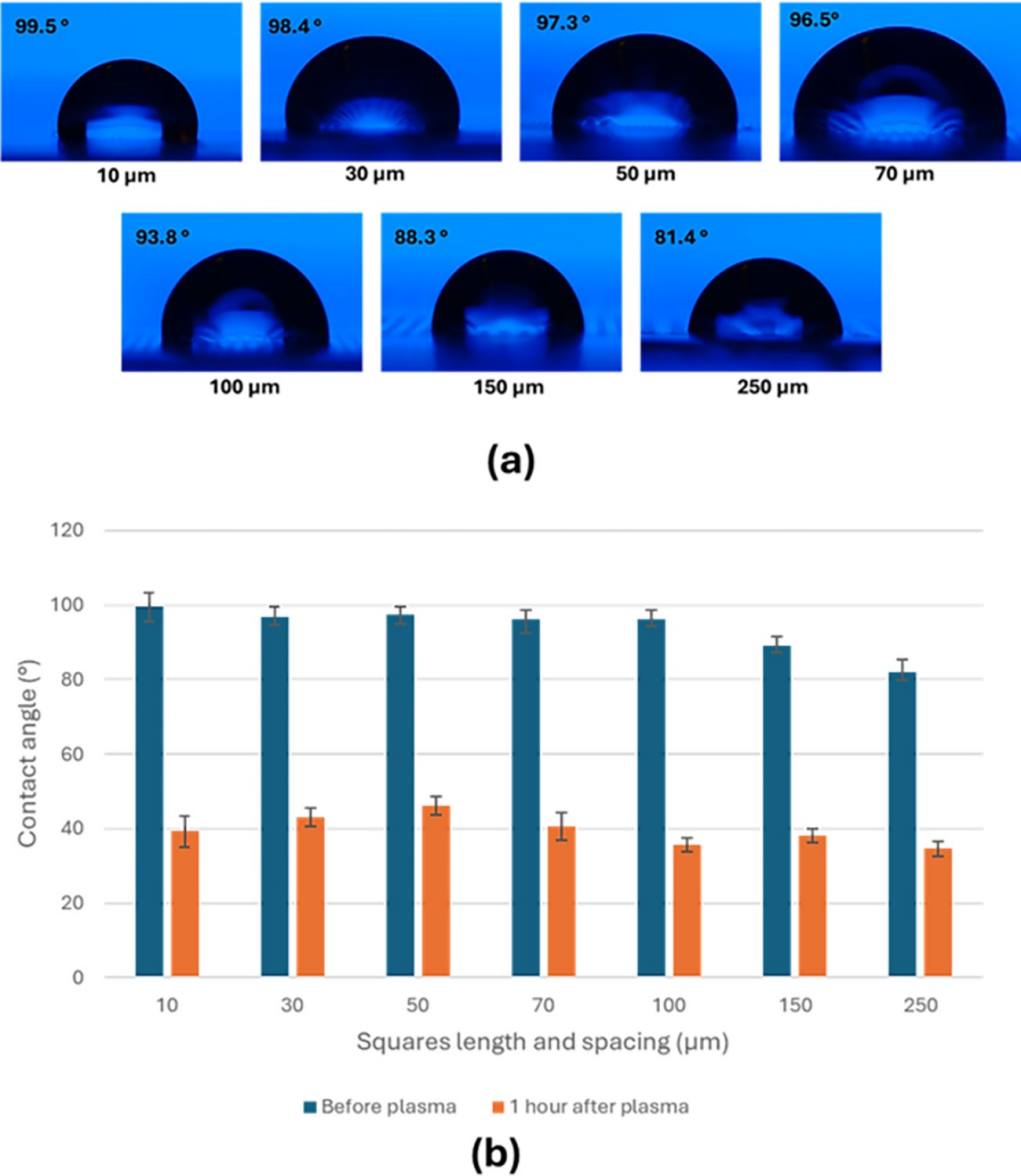
Contact angles for a symmetrical design (squares). Photographs depict DI droplets on the surface of different spacing and sizes (a). Contact angle measurements before and after plasma treatment (b).

The wettability of the square-shaped patterns was assessed by measuring the contact angle, revealing consistent contact angles from all directions due to their symmetry, as depicted in **Fig 4A**. Interestingly, the contact angle exhibited minimal variation with changes in square length and spacing, as illustrated in **Fig 4A.** Specifically, square sizes of 10, 30, 50, 70, and 100 μm displayed hydrophobic behavior with contact angles measuring 99°, 97°, 97°, 96°, and 96° ± 4°, respectively. However, for larger sizes, such as 150 and 250 μm in length and spacing, the contact angles reduced to 89° and 82° ± 4°, respectively, indicating a transition towards weak hydrophilic behavior.

The impact of plasma treatment on surface wettability of patterned surfaces was examined in this study. Following plasma treatment, the surfaces were initially assessed one hour later, revealing a substantial decrease in contact angle across all designs. For instance, the contact angle decreased from 99° to 39° ± 4° for the 10 μm square length, indicating a shift from hydrophobic to hydrophilic behavior induced by the plasma treatment. Similar trends were observed for all square sizes and spacings, as demonstrated in **Fig 4B**.

### Lines array design (non-symmetrical topography)

The second design comprises parallel lines, with the line width identical to the spacing between adjacent lines. This study explores how varying the spacing between the lines affects wettability and Candida attachment. **Fig 1B** presents a schematic diagram of the lines design topography, while **Fig 2** illustrates the fabrication method. Six different gap sizes were examined, ranging from 30 to 250 μm in line width and spacing. **Fig 3C** and **3D** present SEM images of the surfaces patterned with lines, showcasing spacings of 30 μm and 150 μm, respectively.

**Fig 5:**
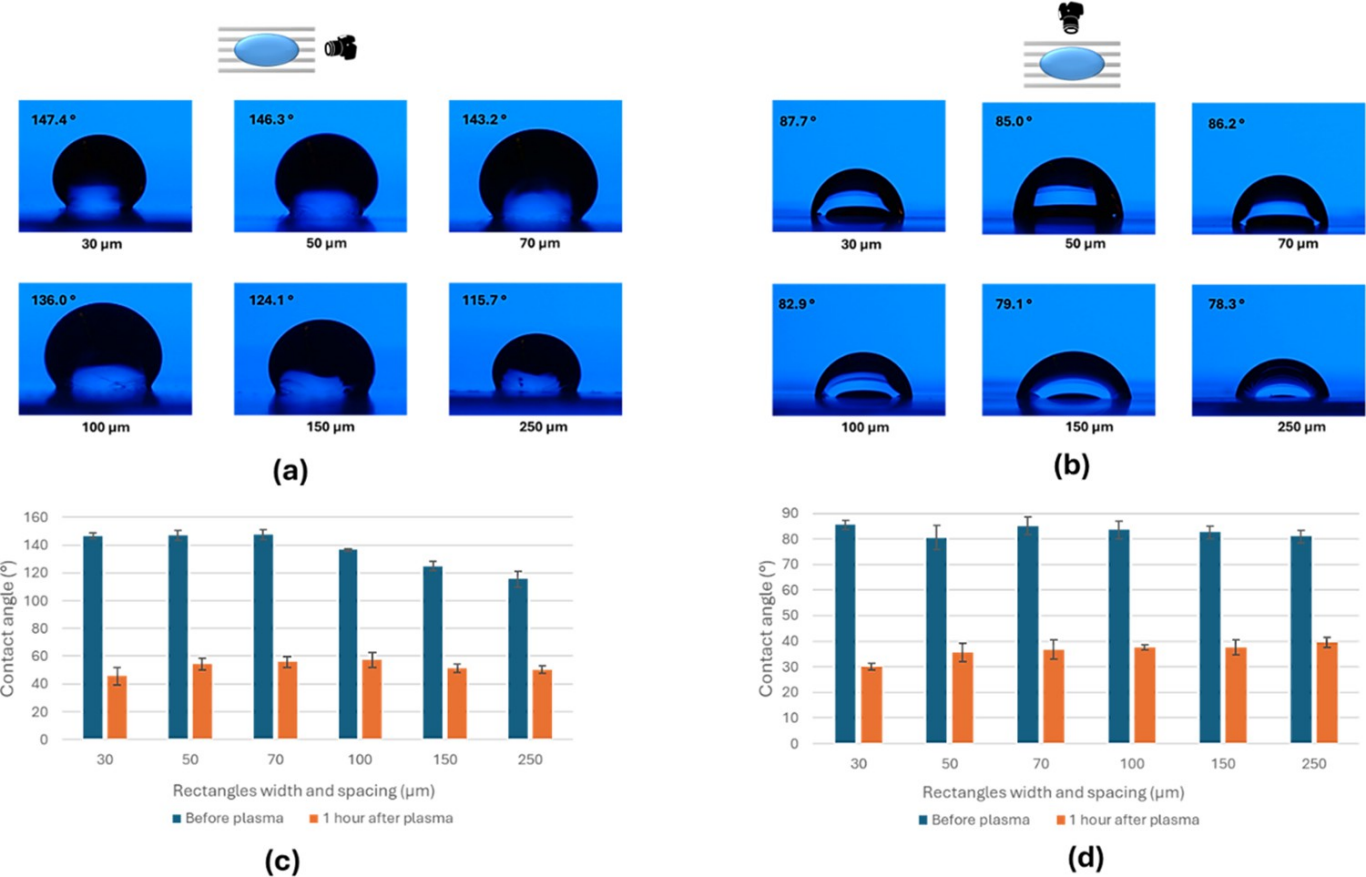
Contact angles for a non-symmetrical design (lines). Photographs depict DI droplets on the surface observed along two orientations: (a) along the lines and (b) perpendicular to the lines. Contact angle measurements before and after plasma treatment are taken along the lines (c) and perpendicular to the lines (d). The insets on top of parts a and b show the direction of measurement for the contact angle.

The non-symmetric pattern was observed to exhibit directional wettability depending on the direction of observation. When the camera was positioned to observe the droplet along the lines, the contact angle was higher compared to when the camera was observing from a direction perpendicular to the lines, as shown in **Fig 5A** and **5B**. Before applying plasma treatment to the surface, the contact angles along the lines were 147°, 147°, 148°, 136°, 125°, and 115° ± 4°, while the angles perpendicular to the lines were 85°, 80°, 85°, 84°, 83°, 81° ± 4° for the 30, 50, 70, 100, 150, 250 μm lines size and spacing, respectively.

After the patterned surface was exposed to plasma, the surface’s wettability was tested one hour after the treatment. The results indicated a significant reduction in the contact angle for all designs. For example, the contact angle decreased from 147° to 45° for the 30 μm line width and spacing when observed from a direction along the line, demonstrating that the plasma treatment caused the surface to change from almost superhydrophobic to hydrophilic. Similar results were observed for all line sizes and spacing, as illustrated in **Fig 5C** and **5D**.

### Temporal evolution of surface wettability

**Fig 6:**
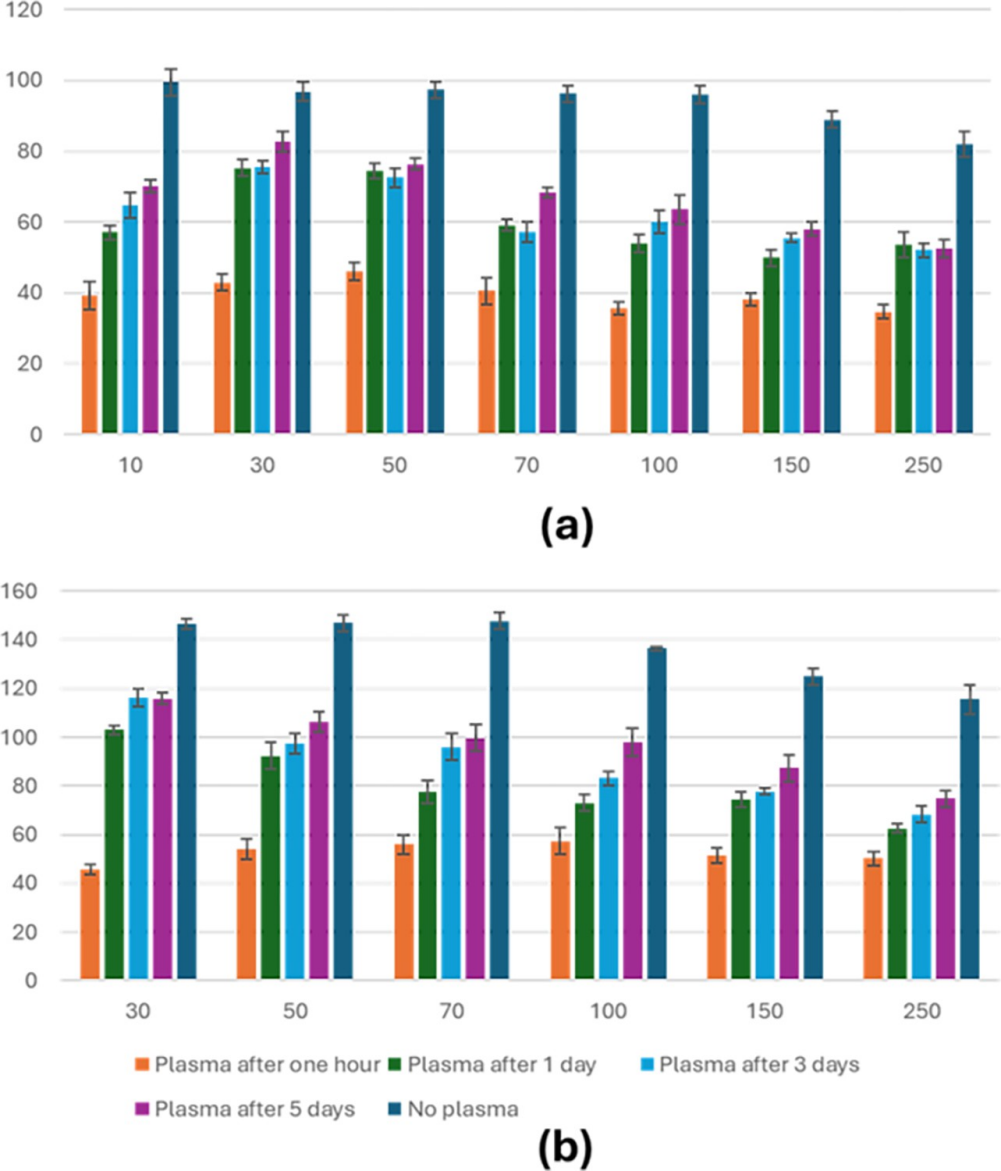
Temporal evolution of surface wettability for both symmetrical and non-symmetrical topographies. (a) Rectangular topography was observed along the lines. (b) Square-shaped topography.

Both symmetrical and non-symmetrical topographies were examined to investigate the evolution of surface wettability over time following plasma treatment. Contact angles were measured at intervals over five days post-plasma treatment, precisely at 1 hour, one day, three days, and five days after treatment, and compared to untreated surfaces. **Fig 6A** displays the results for symmetrical topographies (squares), while **Fig 6B** shows the results for non-symmetrical topographies (lines observed along the lines). The contact angle of the line’s topography also exhibited a similar reduction in wettability along the other direction. The findings indicate a gradual decline in surface wettability over time for both topographical types. By day 5, the contact angles for all designs increased, reaching a saturation point that was not equal to that of untreated samples.

### Candida biofilm formation on the produced surfaces

**Fig 7:**
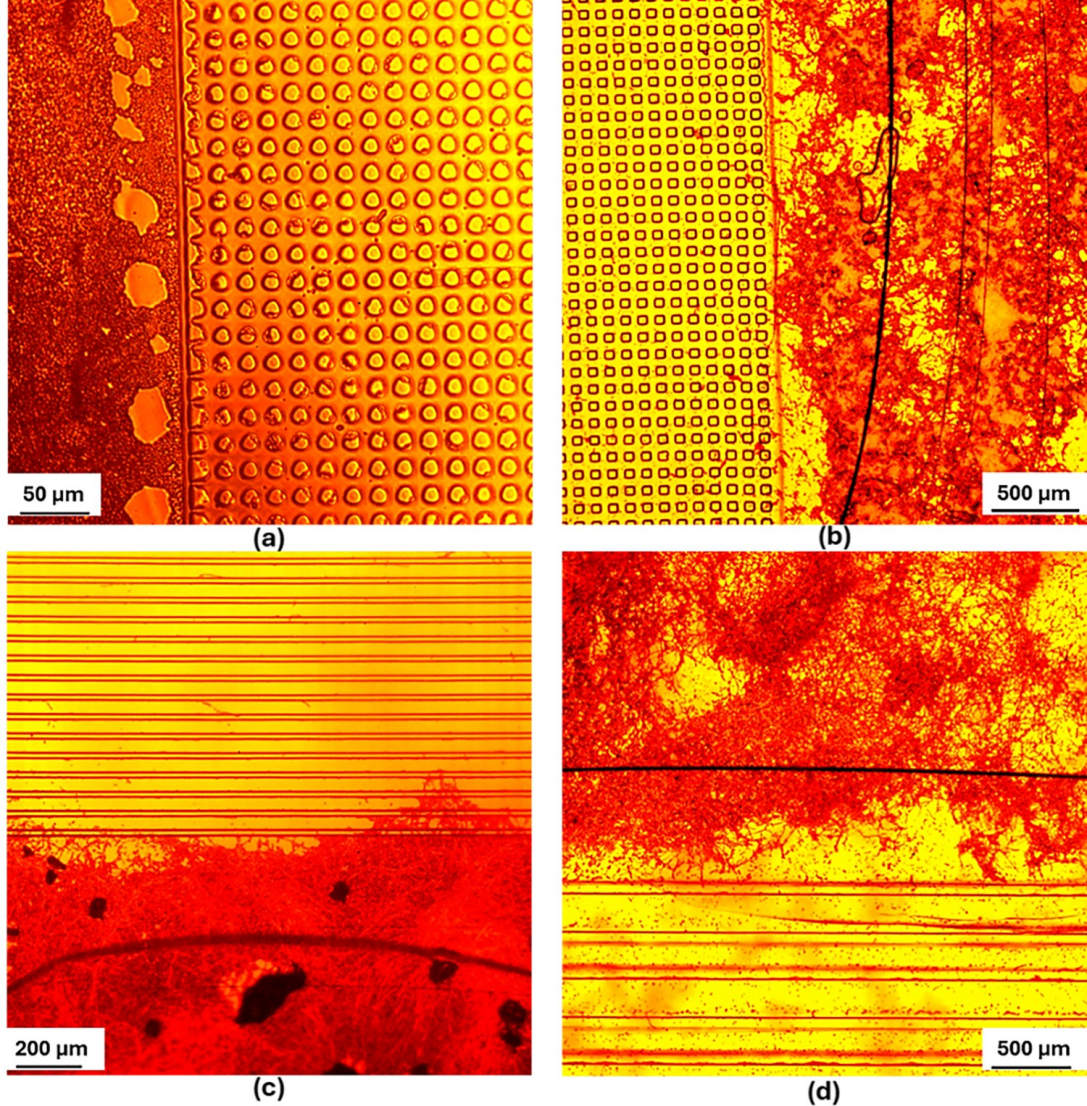
Candida biofilm formation on symmetrical and non-symmetrical topographies. (a) 10 μm squares, (b) 30 μm squares, (c) 30 μm lines, and (d) 250 μm lines.

To explore the influence of surface topography on biofilm formation and attachment, we cultured the DK318 strain of *C*. *albicans* on patterned surfaces with both topographies following plasma treatment. Untreated samples were known to be hydrophobic, suggesting no candida attachment due to surface wettability, as supported by previous study findings [11]. To simulate the sterilization process of indwelling devices using UV plasma and eliminate wettability effects, candida was cultured on both topographies post-plasma treatment. Based on the results in **Fig 6**, the surfaces were expected to be hydrophilic, and candida attachment was anticipated if the surface was flat, as per previous findings. However, both topographies, as shown in **Fig 7**, prevented Candida biofilm formation on the surface. Square patterns with a size of 10 μm almost entirely inhibited biofilm attachment and formation, whereas very few Candida cells were observed between and on top of the squares in the case of 50 μm spacing and length, as shown in **Fig 7A**and **7B**. Conversely, for lines sized at 250 μm, more Candida attachments were observed on top and between the lines compared to those sized at 30 μm, as illustrated in **Fig 7C** and **7D**.

The Candida biofilm growth was quantified through image analysis for Candida cultures on flat surfaces and surfaces with patterned topographies. This analysis involved grayscale image analysis with ImageJ software [21]. Ten images were captured for each case, and the mean intensity value and standard deviation were calculated.

The results presented in **S1 Fig in S1 File**, reveal significant differences in mean intensity due to surface treatments. Biofilms on patterned surfaces exhibited significantly different mean intensities within both line patterns (p = 0.0008372) and square patterns (p = 0.02439). Interestingly, the shape of the pattern (line or square) did not significantly affect the mean intensity (p = 0.4273). **S1 Fig in S1 File**, a boxplot, visually summarizes these findings, providing a clear overview of the data distribution across different surface treatments. These results underscore the influence of surface topography on Candida biofilm growth and highlight potential applications in both biomedical and industrial contexts.

## Discussion

Microbial biofilms represent a significant challenge in persistent human infections due to the increased antimicrobial resistance [22, 23]. Consequently, infections associated with biofilms on retained indwelling medical devices may reoccur once antibiotic treatment is stopped, potentially requiring the removal of the device [24].Therefore, medical device surface treatment to resist attachment and biofilm formation is gaining momentum in the field.

For example, Graham et al. studied the effect of introducing lines and holes rough patterns to the PDMS on cellular attachment [25]. They showed that all patterned surfaces exhibited a notable decrease in cell attachment under static conditions compared to the smooth surface. Arrays of holes were found to be more efficient in minimizing cell attachment. Francone et al. also fabricated two hierarchical pattern designs on Polypropylene (PP) to study the effect of these patterns on the bacteria adhesion [26]. In their first design, the nanostructure was patterned on top of the microstructure pattern, while in the second design, the nanostructure was patterned at the bottom of the microstructure pattern. The first design exhibited a contact angle of 146.9°, while the contact angle for the second pattern was 133.4°. Both designs showed a high capability of reducing cell attachment. However, the second design showed a consistent reduction in cell attachment with both bacteria tested, *E*.*coli* and *S*.*aureus*. Previously, we investigated the impact of surface wettability on *C*. *albicans* biofilm formation [11]. The wettability of COC surfaces was modified through plasma treatment, reducing the contact angle from 110° to 40°. Both treated and untreated surfaces were then subjected to *C*. *albicans* growth experiments. The results revealed that the hydrophilic regions of the treated COC surface showed significant *C*. *albicans* biofilm growth and attachment, while the hydrophobic areas inhibited attachment.

Given that many medical devices undergo plasma sterilization, this leads to increased surface hydrophilicity and subsequent biofilm attachment. By modifying the surface topography, this study presents a novel approach to impede *C*. *albicans* attachment and biofilm formation on medical devices, even on hydrophilic surfaces. A photolithography process was used to create two distinct surface topographies fabricated with symmetrical and non-symmetrical patterns (lines and squares).

First, the proposed strategy involves altering the surface topography to prevent biofilm formation. The investigation focused on lines and squares topographies microfabricated with varying sizes using photolithography. Swelling the substrates in an alkane hydrocarbon (decane) was employed to create both topographies. Next, the wettability of both topographies was assessed after plasma treatment to confirm hydrophilicity across all surfaces. After one hour post-plasma treatment, all surfaces in both topographies became hydrophilic, exhibiting low contact angles, as shown in **Fig 6**. The evolution of surface wettability over time showed a significant impact, with wettability increasing after one day and reaching saturation after almost five days. Biofilm formation and candida attachment were observed only in flat, unpatterned areas. A substantial amount of *C*. *albicans* biofilm was evident on the COC surface, consistent with expectations due to its hydrophilic nature. In contrast, both symmetrical and non-symmetrical topographies, though hydrophilic, showed no biofilm formation. The observed variations in mean intensity values suggest that Candida biofilm production differs across the tested surfaces, as illustrated in S1 Fig in S1 File. Compared to flat surfaces, the higher mean intensity on patterned surfaces indicates a potential acceleration in biofilm formation. This phenomenon may be attributed to the impact of surface topography on early adhesion and subsequent biofilm maturation. The standard deviations reflect the diversity within each condition, further emphasizing the consistency and repeatability of our experimental measurements.

## Conclusion

The research demonstrated that altering the surface topography effectively inhibited biofilm formation by *C*. *albicans*, offering a promising strategy for mitigating *Candida*-associated infections on medical devices. Despite the surface’s hydrophilicity after plasma treatment, which is known to promote biofilm attachment, the patterned areas remained resistant to biofilm formation. These findings emphasize the potential of surface topography and wettability to enhance the biocompatibility of medical devices and reduce the risk of biofilm-related infections. Overall, this study provides valuable insights into the interplay between surface topography, wettability, and biofilm formation, opening up promising avenues for future research and development in medical device design and infection prevention strategies.

## Supporting information

S1 File (DOCX)

## List of abbreviations

DEG: Differentially Expressed Genes
GO: Gene Ontology
FDR: False Discovery Rate
PCA: Principal Component Analysis
WT: Wild-Type
GC: MS Gas chromatography-mass spectrometric
YEPD: Yeast extract-peptone-dextrose
HISAT: Hierarchical Indexing for Spliced Alignment of Transcripts
