# Supplementary material for: Modification of surface topographies to inhibit candida biofilm formation
Source: PLoS One. 2024 Oct 28;19(10):e0308705. doi: 10.1371/journal.pone.0308705 (PMC11515951; doi:10.1371/journal.pone.0308705)
Supplement: S1 File — (DOCX) [file pone.0308705.s001.docx]

**S1 File**

The *Candida* biofilm growth was quantified through image analysis for *Candida* cultures on flat surfaces and surfaces with patterned topographies. This analysis involved grayscale image analysis with ImageJ software [1]. Ten images were captured for each case, and the mean intensity value and standard deviation were calculated.

The results revealed significant differences in mean intensity due to treatments: biofilms on patterned surfaces showed significantly different mean intensities within both line patterns (p-value = 0.0008372) and square patterns (p-value = 0.02439). Interestingly, the shape of the pattern (line or square) did not significantly affect the mean intensity (p-value = 0.4273). Fig S1, a boxplot, visually summarizes these findings, providing a clear overview of the data distribution across different surface treatments. These findings underscore the influence of surface topography on Candida biofilm growth and highlight potential applications in biomedical and industrial contexts.

Top of Form

Bottom of Form


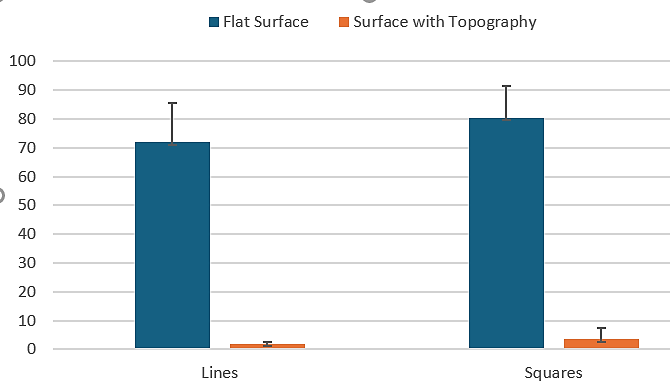


Fig S1. Quantification of Candida Biofilm Growth on Patterned Surfaces Using Grayscale Image Analysis

1. R. WS, "Imagej, us national institutes of health, bethesda, maryland, usa," [*http://imagej*](http://imagej)*. nih. gov/ij/,* 2011.
